# Novel Genome-Engineered *H* Alleles Differentially Affect Lateral Inhibition and Cell Dichotomy Processes during Bristle Organ Development

**DOI:** 10.3390/genes15050552

**Published:** 2024-04-26

**Authors:** Tanja C. Mönch, Thomas K. Smylla, Franziska Brändle, Anette Preiss, Anja C. Nagel

**Affiliations:** 1 Department of Molecular Genetics, Institute of Biology, University of Hohenheim, 70599 Stuttgart, Germany; tanja.moench@uni-hohenheim.de (T.C.M.); thomas.smylla@noscendo.com (T.K.S.); franziska.braendle@bc.biol.ethz.ch (F.B.); 2 Institute of Biology, University of Hohenheim, 70599 Stuttgart, Germany; a.preiss@uni-hohenheim.de

**Keywords:** *Drosophila melanogaster*, bristle organ development, genome engineering, Notch antagonist Hairless, Notch signaling pathway, Notch repression, repressor complex, Suppressor of Hairless

## Abstract

Hairless (H) encodes the major antagonist in the Notch signaling pathway, which governs cellular differentiation of various tissues in *Drosophila*. By binding to the Notch signal transducer Suppressor of Hairless (Su(H)), H assembles repressor complexes onto Notch target genes. Using genome engineering, three new *H* alleles, *H^FA^*, *H^LLAA^* and *H^WA^* were generated and a phenotypic series was established by several parameters, reflecting the residual H-Su(H) binding capacity. Occasionally, homozygous *H^WA^* flies develop to adulthood. They were compared with the likewise semi-viable *H^NN^* allele affecting H-Su(H) nuclear entry. The *H* homozygotes were short-lived, sterile and flightless, yet showed largely normal expression of several mitochondrial genes. Typical for *H* mutants, both *H^WA^* and *H^NN^* homozygous alleles displayed strong defects in wing venation and mechano-sensory bristle development. Strikingly, however, *H^WA^* displayed only a loss of bristles, whereas bristle organs of *H^NN^* flies showed a complete shaft-to-socket transformation. Apparently, the impact of *H^WA^* is restricted to lateral inhibition, whereas that of *H^NN^* also affects the respective cell type specification. Notably, reduction in *Su(H)* gene dosage only suppressed the *H^NN^* bristle phenotype, but amplified that of *H^WA^*. We interpret these differences as to the role of H regarding Su(H) stability and availability.

## 1. Introduction

**Figure 1:**
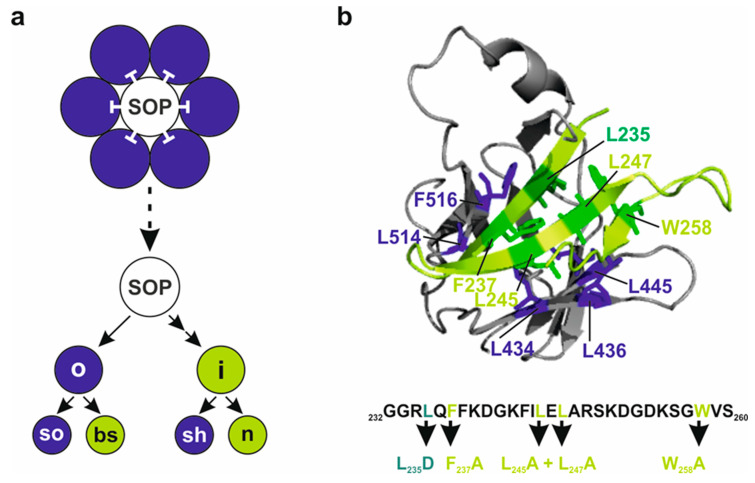
Scheme of Notch activity and Su(H) molecular structure. (**a**) Schematic sensory organ lineage. By activating the Notch signaling pathway, the sensory organ precursor (SOP, white) inhibits the surrounding cells (blue) from becoming committed to the neuronal fate. Asymmetric divisions lead to inner (i) versus outer (o) cell fate, to bristle shaft (bs) versus bristle socket cell (so), and to sheath cell (sh) versus neuron (n). Signal-sending cells are shown in green; cells receiving Notch signaling activity are shown in blue. (**b**) Relevant part of the Su(H)-H complex shown as ribbon model, zooming into interacting domains: H CSL-ID (light green, mutated amino acids in darker green) and Su(H) CTD (grey, interacting amino acids in dark blue). Numbers refer to amino acid position in the respective protein (PDB-ID 5E24). Below, relevant amino acid sequence of the H CSL-ID, highlighting the novel amino acid replacements in light green.

The various cell types of higher animals are determined during development by the activity of specific transcriptional regulators that change cell fate according to external signals. For instance, signals may derive from the Notch signaling pathway, which belongs to a small number of highly conserved signaling pathways governing cell fate and development (reviewed in [1,2,3]). Thereby, the Notch pathway allows the direct communication of neighbouring cells. The underlying principles have been elucidated previously using genetic and molecular approaches in the model organism *Drosophila melanogaster* (reviewed in [3,4]). In *Drosophila*, Notch activity governs a plethora of developmental events, including neurogenesis, myogenesis, cardiogenesis, hematopoiesis and oogenesis. Moreover, the Notch signaling pathway is instrumental for the correct differentiation of various stem cells including the germ line, the adult nervous system and the gut (reviewed in [3,5,6,7,8,9]). Therefore, defects in Notch pathway components are characterized by notoriously pleiotropic phenotypes. Mutations affecting the Notch receptor itself, its ligands or signal transduction components including transcriptional regulation, cause recessive lethality and frequently induce haplo-insufficient dominant phenotypes, e.g., affecting wing and bristle development in the adults [3,4,10]. A prime example is the selection of the mechano-sensory organ precursor (SOP) from a cluster of cells with primary neuronal fate in a process called ‘lateral inhibition’ (Figure 1a). Here, the presumptive SOP inhibits the adjacent cells from becoming neuronal by activating the Notch pathway, thereby forcing them into epidermal fate. Subsequent asymmetric cell divisions of the SOP eventually give rise to the outer cell types, bristle shaft and socket, and the inner cell types, neuron and sheath cell, due to differential Notch activity (Figure 1a) [11,12,13]. Ultimately, the fly’s body is decorated with large bristles (macrochaetae) at fixed positions and with evenly distributed small hairs (microchaetae) [11,14]. Accordingly, variations in Notch activity result in different outcomes. In terms of the SOP, reduced Notch activity leads to an increase in bristle numbers, whereas Notch activity gain precludes neuronal development of the SOP, resulting in a loss of the entire bristle organ. In contrast, aberrant Notch activity changes during the subsequent lineage decisions causes cell type transformations, most easily visible for the outer cells: a gain of Notch activity causes a shaft-to-socket transformation resulting in a double socket phenotype, whereas a loss of Notch activity induces the opposite, namely duplicated bristle shafts (reviewed in [3,15]). Lateral induction of Notch activity via differential ligand presentation, however, governs the formation of the presumptive wing margin [2,3]. A failure of this process causes notched wings, as seen in the adult heterozygous *Notch* mutant flies [2,3,16]. Similar principles of lateral induction/inhibition are also observed in other contexts like the formation of wing veins that receive their final width by Notch activity [5,17]. While reducing Notch activity causes wing vein broadening, a gain of Notch induces thinning or loss of veins [17].

The Notch signaling pathway was named after the Notch receptor, which upon activation by its ligand is cleaved to release the Notch intracellular domain NICD into the cytoplasm. There, NICD binds to its transcription factor named Suppressor of Hairless (Su(H)) in *Drosophila*, abbreviated to CSL in vertebrates (reviewed in [1,2,3]). Together with additional factors, they assemble a transcriptional activator complex onto Notch target gene promoters [18,19]. Su(H) is a tremendously well-conserved DNA-binding protein amongst higher eukaryotes [19,20,21]. Like its homologues, Su(H) is characterized by three structural domains: the N-terminal domain (NTD), the β-trefoil domain (BTD) and the C-terminal domain (CTD). NTD and BTD bind to the DNA, while BTD and CTD mediate contact with NICD during activator complex assembly [19,22].

In the absence of Notch receptor activation, Notch target genes are silenced by a repressor complex formed by Su(H), Hairless(H) and further corepressors in *Drosophila*. H contacts exclusively the CTD of Su(H) through an unconventional binding conformation with an exceptionally high affinity at nanomolar range [23,24,25]. The interaction domain of H (CSL-ID) forms a β-hairpin that inserts deeply into CTD’s hydrophobic core, contacting ten amino acids through purely hydrophobic interactions (Figure 1b) [25]. Primary contact sites in Su(H) are three Leucine residues at position 434, 445 and 514 and a Phenylalanine at 516. Five amino acids in the CSL-ID of H, Leucine 235, 245 and 247, Phenylalanine 237 and Tryptophan 258, provide a hydrophobic surface to the β-hairpin when buried into CTD’s core (Figure 1b) [25]. The consequential distortion of Su(H)’s CTD conformation precludes NICD binding, i.e., formation of activator and repressor complexes is mutually exclusive [25]. Hence, Su(H) acts as a molecular switch, as activator or repressor, depending on the recruited cofactors. Notably, Su(H)’s cofactors play additional and unexpected roles. Firstly, both NICD as well as H carry Su(H) into the nucleus, i.e., its primary site of action, which is similar to mammals, where CSL is likewise co-imported by either NICD or corepressors [26,27]. Secondly, the stability of *Drosophila* Su(H) depends on protein complex formation with its cofactors, NICD and H [28,29].

By way of genome engineering in *D. melanogaster*, we generated several specific alleles of *Su(H*) as well as of *H* to study the structure–function relationship of the repressor complex [28,30]. Mutations in the *H* gene were created to show, for example, that two characteristic protein isoforms of H are derived from differential translation initiation [31]. Moreover, several amino acid replacements allowed us to destroy H’s essential nuclear localization signal NLS3, thereby demonstrating its requirement for H as well as for Su(H) nuclear entry [27]. Likewise, the additional mutation of the adjacent nuclear export signal (NLS3NES) showed that H nuclear shuttling is instrumental to its function [27]. In fact, whereas the H^*NLS3^ allele was indistinguishable from an *H^attP^* null allele, the double mutant H^*NLS3*NES^ (hereafter abbreviated to *H^NN^*), retained H activity: it allowed some nuclear Su(H) protein accumulation, and showed milder phenotypes including rare homozygous survivors [27].

Genome engineering also helped to confirm the X-ray structure predictions for the H-Su(H) repressor complex [25,28]. For example, the replacement of the Leucine residues L434, L445 and L514 with Alanine in Su(H)’s CTD ((*SuH*)*^LLL^*) completely abolished the binding to H [25,28]. Moreover, in the *H^LD^* mutant the exchange of Leucine 235 for Aspartate completely disrupted H-Su(H) binding [24,25,28]. The negative charge introduced in the CSL-ID abolished the hydrophobic interactions necessary for the binding, explaining the failed complex formation; however, this added little to the understanding of the repressor complex structure [25]. We aimed at introducing conservative changes to those amino acids predicted important for the binding by structural analyses. To this end, we replaced amino acids F237, L245 and L247, as well as W258, with Alanine in the CSL-ID of H and assayed the binding properties and remaining biological activity of the respective mutants in overexpression assays [32] (Figure 1b). We expected a linear decrease in the activity of the H protein variants, reflecting their residual Su(H) binding capacity as determined by prior isothermal titration calorimetry [25]. Unexpectedly, the three mutant variants (*H^FA^*, *H^LLAA^* and *H^WA^*) behaved very similarly in overexpression analyses, indicating that all three had lost most of their H repressive activity [25,32]. Their residual binding to Su(H), however, was uncovered by a combined overexpression of the H mutant variants together with Su(H), causing phenotypes similar to the wild-type H isoform [32]. Apparently, a subtle weakening of the cohesion within the H-Su(H) repressor complex was sufficient to disrupt its activity. The overexpression experiments did not allow us to differentiate the specific biological functions of the mutant H proteins.

Therefore, we decided to introduce the respective mutations via genome engineering into the *H* locus to explore the residual function of the generated variants. The results allowed us to establish a phenotypic series based on various parameters, closely reflecting the in vitro binding ability to Su(H), and thus confirming the structure predictions. Whereas the *H* allele *H^FA^* was similar to the *H* null allele *H^attP^*, *H^LLAA^* retained some H activity. *H^WA^* showed the mildest phenotypes; some flies even developed to adulthood. Similar to the *H^NN^* survivors, the *H^WA^* adults showed extreme *H* phenotypes. Both alleles were short-lived, impaired in locomotion and sterile. We noted distinct differences regarding bristle development, however. While shaft-to-socket transformation was nearly complete in *H^NN^*, binary cell fate decisions were not affected in the *H^WA^* homozygotes. We discuss this difference in light of a limited availability of Su(H) protein in the *H^WA^* background.

## 2. Materials and Methods

### 2.1. Fly Work

Flies were maintained on standard fly food (0.8% agar–agar, 8% corn meal, 8% malt extract, 2.2% treacle, 1% soy meal, 1.8% dry yeast and 0.6% propionic acid) in a 12 h light/dark cycle and 80% humidity at 18 °C, and crosses were reared at 25 °C unless stated otherwise, using Oregon R (BL5) and *H^cwt^* [30] as wild-type controls as indicated. We note that *H^cwt^* displays a weak *H* bristle phenotype in trans over *H* null alleles [30]. However, because the respective H* alleles were based on H cDNA, *H^cwt^* was still used as control [32]. For loss of function alleles, *H^attP^*/TM6B (BL94608) [30], *H^LD^*/TM6B (BL93865) [30], *H*^22^/TM6B (BL93661) [33] and *Su(H)^attP^*/CyO (BL93861) [28] were used. We note that *H*^22^ was recessive lethal in our hands, and no living adults were obtained unlike earlier descriptions [33]. For more information, please visit Flybase [34]. Standard genetic techniques were applied for re/combining respective fly stocks.

The alleles *H^FA^*, *H^LLAA^* and *H^WA^* were generated through genome engineering as previously described [30,35]. Mutant H cDNAs [32] were cloned into the pGE-attB^GMR^ vector [35], to be integrated using the *H^attP^* allele as a platform by site-specific recombination as outlined earlier [30,31]. Genotypes were confirmed by PCR and sequencing where applicable. Documentation of mutant phenotypes was performed as described earlier [30,36].

H* mutant alleles were balanced with TM6B balancer, allowing us to distinguish the homo- and hemizygous H* larvae and pupae from heterozygotes thanks to the dominant *Tubby* marker. The usual crosses were set up with 8 males and 15 virgin females. Scanning electron micrographs of uncoated adults were taken with a Neoscope table-top scanning electron microscope (Nikon, Tokyo, Japan). Adult wings, dehydrated in ethanol, were mounted in Euparal (Carl Roth, Karlsruhe, Germany), and documented with an ES120 camera (Optronics, Goleta, CA, USA) mounted to a Zeiss Axiophot (Carl Zeiss, Jena, Germany) with Pixera viewfinder 2.0 software.

### 2.2. Immunohistology

Clonal analysis based on the Flp/FRT technique followed the protocol outlined before [37]. To this end, the respective H* allele, recombined with P{neo FRT}82B (BL2050), was crossed with P{neo FRT}82B P{Ubi-GFPS65Tnls}3R/TM6B (BL32655). FLPase was induced through a 1 h heat shock at 37 °C in early second-instar larvae. Wing imaginal discs were dissected from third-instar wandering larvae in PBS, fixed in 4% paraformaldehyde, washed in PBT (PBS + 10% Tween) and blocked with 4% normal goat serum, before adding primary antibodies (anti-Dpn 1:200, Abcam, Cambridge, UK; anti-GFP 1:250, Santa Cruz Biotech, Dallas, TX, USA).

Pupal nota were dissected from young pupae about 30 h after pupal formation and handled on ice as described above, using PBX (PBS + 0.3% Triton X100) for the washes [38,39]. Primary antibodies were mouse anti-Pros (1:50; MR1A, developed by C.Q. Doe and obtained from DSHB, Iowa City, IA, USA), rat anti-Elav (1:25; 7E8A10, developed by G.M. Rubin and obtained from DSHB), Iowa City, IA, USA and rabbit anti-Su(H) (1:500; Santa Cruz Biotech, Dallas, TX, USA).

Gonads were dissected as outlined before [40] from third-instar wandering larva, from pupae (24 h after pupal formation) or adults (4–5 days old) in PBS, fixed in 4% paraformaldehyde and washed in PBX to be treated with rat anti-vasa (1:20; developed by A.C. Spradling, A.C. and D. Williams, obtained from DSHB, Iowa City, IA, USA), and mouse anti-Hts (1:20; 1B1, developed by H.D. Lipshitz, obtained from DSHB, Iowa City, IA, USA).

After several washes in PBT (wing imaginal discs) or PBX (gonads and nota) and finally PBS, respective secondary goat antibodies (1:200), coupled to FITC, Cy3 or Cy5 were applied (Jackson Immuno-Research laboratories via Dianova, Hamburg, Germany). Fluorescently labelled tissue was mounted in Vectashield (Vector labs, Eching, Germany) and documented with a BioRad MRC 1024 laser scan microscope (Carl Zeiss, Jena, Germany) using Lasersharp 2000TM software.

Nuclei were labelled in fixed tissue with DAPI (1 µg/mL) in PBX for 4 min at room temperature in the dark, before the final washes with PBX and finally PBS. Tissue was mounted in Vectashield (Vector labs, Eching, Germany) or 80% glycerol. Pictures were taken with an EOS 700D camera (Canon, Tokio, Japan) mounted to an Axioskop 2 plus (Carl Zeiss, Jena, Germany). Pictures were assembled using Image J win64, Corel Photo Paint 2018 and Corel Draw 2018 software.

### 2.3. Behavioral Assays

Fertility assay: fertility of both sexes was assayed. To assay male fertility, 10 males of 2–3 days age and 15 wild-type virgins (Oregon R) of 7–10 days age were maintained at 25 °C. Reciprocal crosses were set up accordingly; in this case, female H* mutants were around only 4 days old. The assay was performed in three biological and two technical replicates. From day three on after mating, vials were inspected daily for larval offspring, in which case the respective parents were classified fertile.

Food uptake and larval olfaction: early third-instar larvae 72 h after egg laying were washed in PBS and transferred onto apple juice–agar plates fully covered with blue-colored yeast for one hour. Food uptake was monitored by the blue-colored gut content. As a measure for larval olfaction, individual larvae were place on apple juice–agar plates and the timespan for reaching yeast paste in a 2 cm distance was recorded.

Climbing assay [41]: twenty male flies of each genotype were selected into empty vials of 5 cm diameter, marked at 4 cm and 8 cm height. Flies were allowed to acclimate for twenty minutes. After tapping the flies down, they were allowed to climb for 10 s while their position was recorded using a smart phone camera. Individuals were counted at 0 cm, below 4 cm, between 4 and 8 cm and above 8 cm. Tapping and recording was conducted ten times with a 2 min pause in between. Five biological replicates were taken.

Flight assay: freshly hatched homozygous flies were selected without CO_2_ anesthesia and maintained for two days at 25 °C in a fresh vial to unfold wings. Individuals were kept head down in batches of five in a petri dish 1 m above ground. After lifting the lid, flies were persuaded to fly by tapping on the dish, repeated once for the ones clinging to the dish (according to [42]). The test was repeated for clinging and falling individuals. The number of flying and flightless individuals was recorded.

Courtship assay: courtship of single pairs of 2–3 days old males and 1-week-old virgin females, pooled without anesthesia, was observed for three hours, recording courtship behavior of males and successful mating [43].

### 2.4. Yeast 2-Hybrid Analysis

The yeast 2-hybrid system was applied with EGY48 yeast cells to assay binary protein–protein interactions [44,45], using H proteins fused to the LexA DNA binding domain provided by the pEG202 vector as bait, and Su(H) proteins fused to the B42 transactivation domain as prey as outlined in detail before [44,45]. Binding of the respective proteins reconstitutes the active transcription factor, driving lacZ reporter gene expression from the pSH18-34 vector, visualized by the blue-colored yeast colonies grown on X-Gal plates. Constructs pEG H, pEG H L_235_D, pJG Su(H) and pJG Su(H) L_434_A L_445_A L_514_A have been described before [24,25,46,47]. pJG Su(H) L_436_A L_445_A L_514_A was established by introducing the L_436_A amino acid replacement into the pJG L_445_A L_514_A construct [25,48]. The constructs pEG H F_237_A, pEG H L_245_A L_247_A and pEG H W_258_A were shuttled into full length H from the respective mutant NTCT precursors [32]. All final constructs were sequence-verified.

### 2.5. Western Blots

Protein extracts were derived from brains and imaginal discs of 15 third-instar homozygous larvae of each genotype, homogenized in 60 µL binding buffer (20 mM HEPES pH 7.6, 150 mM MgCl_2_, 10% glycerol, 0.05% NP-40, 1 mM DTT and ROCHE complete ULTRA protease inhibitor mini tablet (Sigma Aldrich, Merck Taufkirchen, Germany). An amount of 50 µL of the homogenate was mixed with 25 µL Blue protein loading dye plus DTT (New England Biolabs, Ipswich, MA, USA); 15 µL was loaded per lane onto a 10% SDS polyacrylamide gel. The blot was sliced along the prestained protein marker (Thermo Fisher Scientific, Waltham, USA), and probed with rat anti-H h5 (1:500) [49] or mouse anti β-tubulin (1:2000) (anti-β-tubulin A7; developed by M. Klymkowsky; obtained from DSHB) and respective AP-coupled goat secondary antibodies (1:1000; Jackson Immuno-Research laboratories via Dianova, Hamburg, Germany).

### 2.6. qRT-PCR

Quantitative RT-PCR was performed on four biological with two technical replicates each, probing homozygous *H^WA^* and *H^NN^* versus control *H^cwt^*. Poly(A^+^) RNA was prepared from 12 third-instar wandering larvae with the PolyATtract^®^ System 1000 (Promega, Walldorf, Germany), followed by a DNase I (RNase free) digest (New England Biolabs, Ipswich, MA, USA). cDNA was synthesized from around 250 ng mRNA using the qScriber cDNA Synthesis Kit (HighQu, via Biozol, Eching, Germany). Real-time qPCR was performed as described before using the Blue S’Green qPCR Kit (Biozym, Scientific GmbH, Hessisch Oldendorf, Germany) on around 5 ng of cDNA and the MIC magnetic induction cycler (bms, via Biozym Scientific GmbH, Hessisch Oldendorf, Germany), including target and no-template controls [40]. Internal reference genes were *cyp33* and *Tbp*. Primer pair sequences are listed at the DRSC FlyPrimer bank [50]: *cnc* (PP60393), *cyp33* (PP14577), *ewg* (PP35080), *GstD1* (PP16044), *mTFB2* (PP26980) and *Tbp* (PP1556). Respective oligonucleotides were obtained from Microsynth (Balgach, Switzerland). The micPCR^®^ software version 12.2 was used for relative quantification of the data, based on REST and taking target efficiency into account [51].

### 2.7. Statistics

Statistical significance was determined by ANOVA using a two-tailed Tukey–Kramer or Dunnett’s approach for multiple comparisons, as indicated. For pairwise comparisons, Student’s *t*-test was applied, presented as highly significant ***, *p* < 0.001; very significant **, *p* < 0.01; significant *, *p* < 0.05; not significant n.s., *p* > 0.05.

## 3. Results

### 3.1. Repressor Complex Formation Addressed by Protein–Protein Interaction Studies and the Generation of Novel H Replacement Mutants Specifically Affecting Su(H) Binding

**Figure 2:**
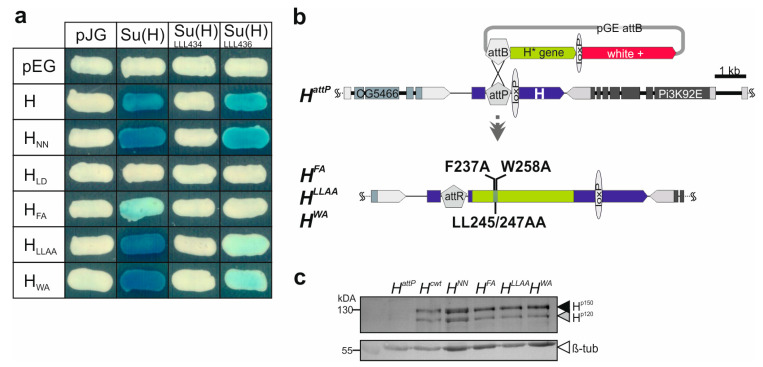
H-Su(H) interaction profile and generation of novel *H* mutants. (**a**) Yeast two-hybrid assay for protein interactions between respective H variants in pEG, and Su(H) variants in pJG vectors. Empty vectors served as controls. Blue coloration indicates protein–protein binding. Constructs encode the following mutants: *H_NN_*, F582A-V585A-L587A; *H_LD_*, L235D; *H_FA_*, F237A; *H_LLAA_*, L245A-L247A; *H_WA_*, W258A; Su(H)_LLL434_, L434A-L445A-L514A; Su(H)_LLL436_, L436A-L445A-L514A. (**b**) Generation of *H* point mutants by genome engineering. Depicted is a scheme of the *H^attP^* locus architecture; it served as a platform for introducing the new H* mutations. Light green, coding region; dark blue, untranslated region. The white^+^ gene (red) served as a marker for the transgenic flies, eventually floxed out with the help of loxP sites. Neighboring genes CG5466 and Pi3K92E are indicated. *H^FA^*, *H^LLAA^* and *H^WA^*, introduced by site-specific integration, resulting in a gene replacement at the endogenous locus. (**c**) H protein expression in homozygous mutant larvae as indicated by Western blot analysis; the typical two H protein isoforms (black arrowhead, H^p150^; grey arrowhead, H^p120^) are detected in larval extracts from homozygotes, except in the null mutant *H^attP^*. Below, β-tubulin (β-tub, white arrowhead) as loading control, derived from the same blot sliced after transfer (see Supplemental Figure S1 for uncropped blot).

Previously, Su(H) protein binding activity of the three constructs *H_FA_*, *H_LLAA_* and *H_WA_* was addressed using a short peptide NTCT overlapping the CSL-ID [32]. The H NTCT peptide, however, binds very strongly, preventing discrimination between the variants. Hence, we introduced the mutations into full-length H protein to re-examine Su(H) protein binding. Wild-type H protein, and *H_LD_* known to completely lack Su(H) binding [24,25], served as positive and negative controls, respectively. In addition, we used *H_NN_* which affects H nuclear translocation [27] but is expected to retain normal Su(H) binding activity. Indeed, binding of *H_FA_* to Su(H) was strongly impaired, whereas *H_LLAA_* and *H_WA_* matched the H and the *H_NN_* controls (Figure 2a) [24,32]. We next tested two different Su(H) variants carrying triple mutations in the CTD, Su(H)_LLL434_ and Su(H)_LLL436_. In Su(H)_LLL434_, three Leucine residues L434, L445 and L514 are mutated to Alanine, completely abolishing any binding to H [25]. Accordingly, no binding was observed to any H variant in the yeast two-hybrid assay (Figure 2a). Su(H)_LLL436_ carries Alanine replacements at L436, L445 and L514, i.e., differs only at position 436 vs. 434 (Figure 1b) [48]. Based on the structure predictions, both Leucine residues contact the CSL-ID [25]; however, L436 lies more peripherally relative to L434, which is positioned rather in the centre of the H-Su(H) interface [25]. We hence may expect some contact of L436 with L247 or W258 in the CSL-ID (Figure 1b). In fact, whereas the binding of Su(H)_LLL436_ to the H and the *H_NN_* controls was only slightly reduced, both *H_LLAA_* and *H_WA_* displayed only a very weak interaction (Figure 2a). We conclude that the singular mutations in the CSL-ID may somehow weaken the interaction to Su(H); however, this becomes visible only upon further impairment of the cohesion on the Su(H) side.

Earlier, we showed that tissue-specific overexpression of either of the three *H* constructs *H^FA^*, *H^LLAA^*, or *H^WA^* had only little impact on fly development, indicative of a lack of Su(H) binding similar to the *H^LD^* construct [32]. In a co-overexpression together with Su(H), however, any of the three was similar to wild-type H, demonstrating their ability to form repressor complexes in the presence of excess Su(H) protein [32]. Taken together, overexpression studies were inconclusive as to the extent the *H* variants retain residual activity. In order to address the question of whether the single- and double-point mutations within the CSL-ID might impair H-Su(H) complex formation in vivo, and hence affect Notch signaling activity, we generated the three alleles *H^FA^*, *H^LLAA^*, and *H^WA^* via genome engineering as outlined before [30,35]. This way, the mutations are introduced into the native locus, granting correct temporal and spatial expression of the respective H* mutant variants. To this end, the mutant H cDNAs were integrated into the endogenous *H* locus by site-specific recombination using the *H^attP^* founder line as a landing platform (Figure 2b). For the subsequent analyses, the resultant alleles were compared with *H^cwt^*, likewise established using the wild-type H cDNA [30]. In order to confirm regular integration and activity of the new *H* alleles, protein expression was analysed in homozygous larvae, demonstrating the presence of the two typical H protein bands [49] (Figure 2c).

### 3.2. Phenotypic Analysis of the Novel H Alleles

**Figure 3:**
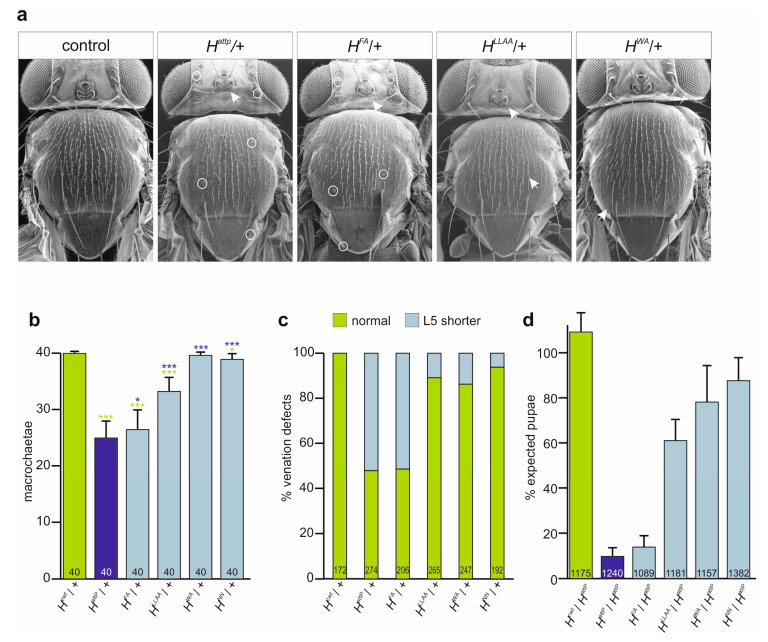
New H* mutant alleles lack H activity to different degrees. (**a**) Scanning electron micrographs of heterozygous H* alleles as indicated uncover the characteristic loss of micro- and macrochaetae (arrows point to examples) and shaft-to-socket transformation (exemplified by circles). (**b**) Number of retained macrochaetae observed in females of the given genotype (n, 40 flies analysed each). *D. melanogaster* adults display 40 macrochaetae on head and notum in fixed positions. For statistical analysis, ANOVA for multiple comparisons according to the Tukey–Kramer method relative to control (green) and *H^attP^*/+ (blue) was employed (*** *p* ≤ 0.001, * *p* ≤ 0.05). (**c**) Wing venation defects characterized by a shortened longitudinal vein L5 observed in the heterozygotes of the given genotype. Total number of evaluated wings indicated in each column. (**d**) Survival rate of given *H* alleles in trans over the null allele *H^attP^* measured as rate of pupae formed relative to the heterozygous siblings. Percentage of expected pupae is according to the mendelian ratio. Three independent experiments were performed. Total numbers of analyzed pupae indicated in each column.

As expected for mutants affecting Su(H) binding, the three *H* alleles affected H activity; however, this was to different degrees. Firstly, the heterozygotes displayed the typical dominant *H* phenotypes, i.e., loss of bristle as well as shortened wing veins (Figure 3a–c) [30,33,52,53,54,55,56]. The defects varied between the alleles, however. Notably, the bristle defects allowed us to establish a phenotypic series *H^attP^* < *H^FA^* < *H^LLAA^* < *H^WA^* < H^+^, which is in agreement with the protein interaction data above and our earlier observations [32] (Figure 3b). All three alleles were homozygous lethal, and died during larval-to-pupal stages, except for *H^WA^* that produced homozygotes at low penetrance (see below). Next, we crossed the mutants to the *H^attP^* null allele and recorded the emergence of the trans-heterozygous offspring. The fraction of observed pupae expected by the mendelian ratio confirmed the above phenotypic series, being lowest for *H^FA^* and highest for *H^WA^* (Figure 3d). Our data so far indicate that *H^FA^* is a very strong *H* allele, albeit not a null mutant, whereas *H^LLAA^*, and even more so *H^WA^*, retain considerable H activity. In these assays, *H^WA^* was indistinguishable from the likewise weak *H^NN^* allele, which has normal Su(H) binding capacity but is impaired in nuclear translocation (Figure 3b–d) [27].

**Figure 4:**
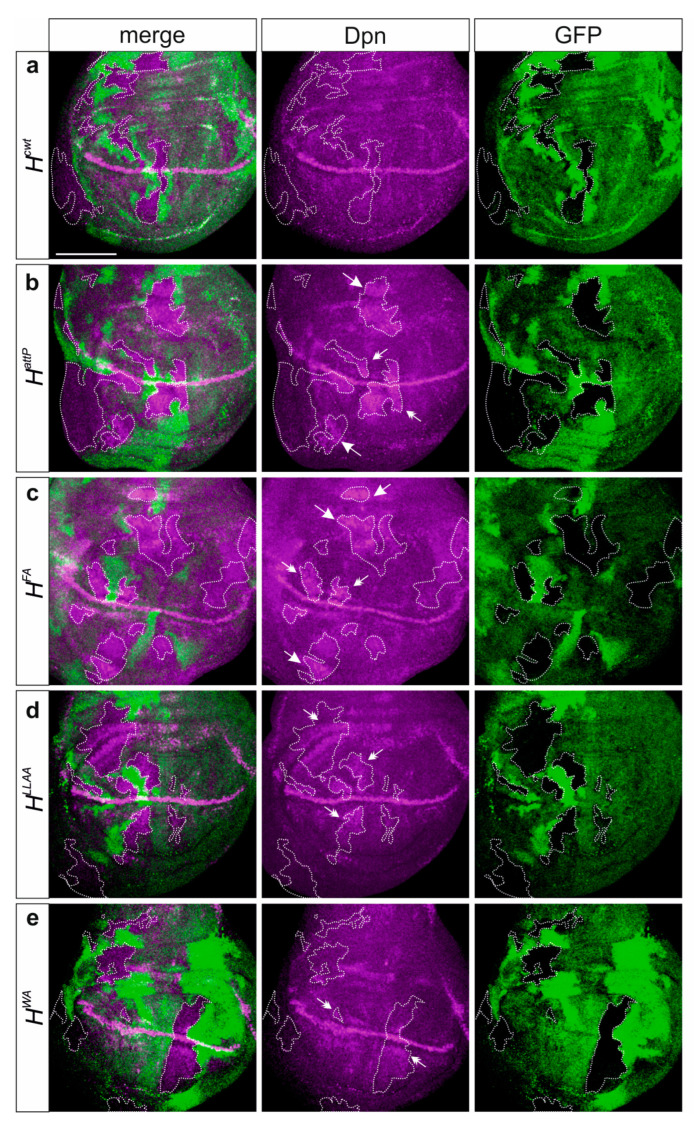
Deregulation of the Notch target gene *dpn* reflects residual activity of the H* mutant alleles. Clonal analysis in the heterozygous alleles (**a**) *H^cwt^*, (**b**) *H^attP^*, (**c**) *H^FA^*, (**d**) *H^LLAA^* and (**e**) *H^WA^*. Expression of Dpn protein (magenta) in wing imaginal discs bearing clones of cells mutant for the respective *H* allele as indicated. The wild type allele carries the GFP marker; hence, homozygous and heterozygous wild-type cells are labelled bright green and light green, respectively, whereas the mutant cells are unlabelled (outlined for clarity). Dpn upregulation in clones is exemplified by arrows outside the Dpn expression domain and by double-headed arrows adjacent to the Dpn expression domain. Size bar, 100 µm in all panels.

By recruiting repressor complexes onto Notch target gene promoters, H acts as major antagonist in the Notch signaling pathway [24,25,57,58,59]. Accordingly, the repression of the Notch target gene *deadpan (dpn)* is abrogated in cells homozygous for the null allele *H^attP^* in most areas of wing imaginal discs (Figure 4a,b) [27,60,61]. A similar de-repression of *dpn* was observed in homozygous *H^FA^* cell clones, whereas *dpn* upregulation was allowed in *H^LLAA^* mutant cells primarily in the vicinity of the normal expression domain (Figure 4c,d). In contrast, *H^WA^* mutant cells were largely normal with regard to *dpn* expression, and only the normal pattern seemed enhanced in some areas, notably along the dorso-ventral boundary (Figure 4e). In sum, the observed phenotypes allowed us to establish a phenotypic series with *H^FA^* being the strongest allele, *H^LLAA^* an intermediate and *H^WA^* the weakest allele in accordance with the residual Su(H) binding activity of the respective mutant proteins.

### 3.3. Homozygous H^WA^ and H^NN^ Alleles Display Strong H Loss of Function Phenotypes and Are Defective in Locomotion

**Figure 5:**
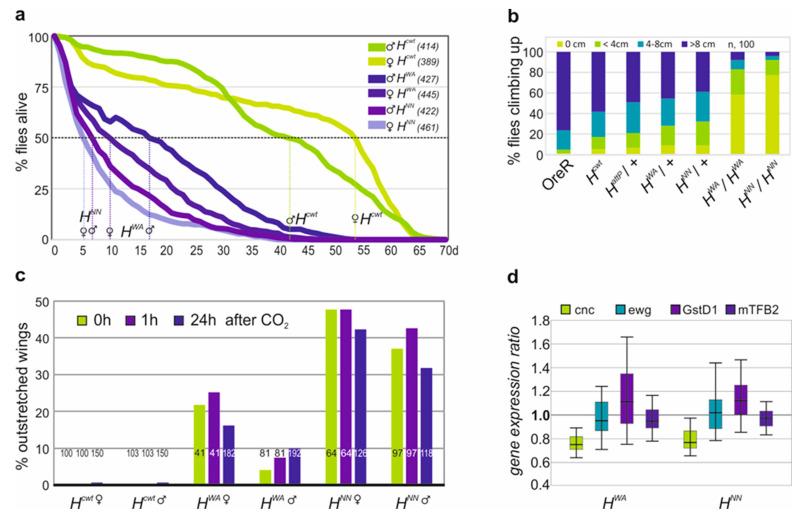
Homozygous *H* mutant alleles are short-lived and display locomotor defects. (**a**) Survival of sexed adults as indicated at 25 °C over the time of 70 days; survivors are shown as a fraction of the total (given in the respective legend). On average, 50% of the control *H^cwt^* females live 54 and males 42 days, whereas *H^WA^* females live only 10 and the males 17 days, and *H^NN^* females 5 and males 6 days. (**b**) Climbing assay: fraction of male flies of the given genotype reaching the indicated height within 10 s (n, 100). (**c**) Outstretched wings observed in control and mutant flies as indicated without CO_2_ anaesthesia, or 1 h or 24 h thereafter as a fraction of the total (indicated within each column). (**d**) Expression levels of *cnc*, *ewg*, *GstD1* and *mTFB2* quantified by qRT-PCR in *H^WA^* and *H^NN^* mutant larvae relative to *H^cwt^* control. Reference genes, *Tbp* and *cyp33*. Bar shows 95% confidence, median corresponds to expression ratio. Amplification efficiencies for *cnc* (0.92), *ewg* (0.94), *GstD1* (0.89), *mTFB2* (0.92), *Tbp* (0.90) and *cyp33* (0.91) were accounted for in determining relative quantities by REST. Unlike *cnc*, which was reduced by 25% in the homozygous mutant larvae *H^WA^* and *H^NN^*, all the others were not significantly altered compared to *H^cwt^*.

The *H^WA^* allele is homozygous semi-viable, allowing us to further study the effects of a strong *H* loss of function. It has been compared with the likewise semi-viable allele *H^NN^* [27]. Both homozygous *H* mutant alleles were short-lived, as described earlier for recessive *H* alleles [33,54]. Half of the *H^NN^* homozygotes lived only around 5 days, and the *H^WA^* homozygotes 10–15 days, whereas the *H^cwt^* control lived more than four times longer (42–54 days) (Figure 5a). Feeding behaviour of the larvae, however, was indistinguishable from the control. The homozygous *H^WA^* and *H^NN^* flies were lethargic, barely moving or climbing, unlike their heterozygous siblings or control flies (Figure 5b). They displayed strong wing venation phenotypes with large gaps in longitudinal veins L4 and L5 (Supplemental Figure S2). Both homozygotes were flightless. Moreover, 10–20% of *H^WA^* and around 40% of *H^NN^* homozygotes displayed an outstretched wing phenotype, which was independent of CO_2_ anesthesia or sex (Figure 5c; Supplemental Figure S3) [62]. As the indirect flight muscles appeared grossly normal, we investigated mitochondrial gene activity, which is crucial for a functional musculature. We concentrated on *erect wing (ewg)*, the *mitochondrial transcription factor B2 (mtTFB2)*, *Glutathione S transferase D1* (*GstD1)* and *cap’n collar (cnc)*. Ewg is important for the biogenesis of mitochondria and the maintenance of the indirect flight muscle [63], whereas mtTFB2 regulates mitochondrial transcription and replication [64]. The two other proteins are important in the oxidative stress response: *cnc* encodes a transcription factor involved in oxidative stress regulation, while the enzyme GstD1 protects from ROS generated during respiration [65,66]. In fact, GstD1 is reduced in a specific *Pten^5^* allele displaying a similar outstretched wing phenotype related to mitochondrial defects [42]. Transcription levels of the four genes were assayed by qRT-PCR (Supplemental Figure S4). Expression of the genes appeared similar in the homozygous mutants relative to the *H^cwt^* control, apart from *cnc*, which was slightly reduced (ca. 25%) (Figure 5d). Based on these results, major defects in flight musculature function seem unlikely, suggesting different underlying problems for example structural defects in the wing hinge or defective flight muscle innervation [67,68].

### 3.4. Fertility Is Impeded in the Homozygous H^WA^ and H^NN^ Alleles

**Figure 6:**
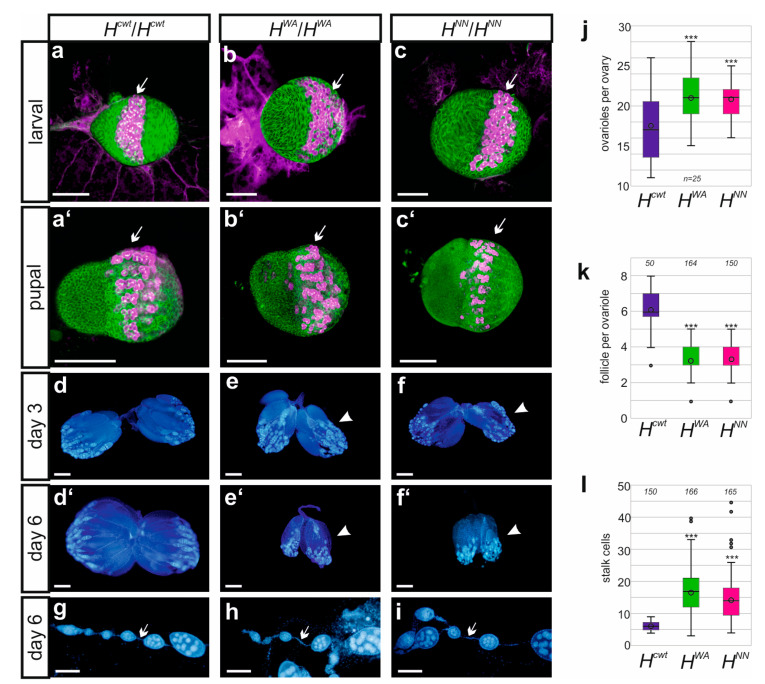
Ovary development is normal early on. (**a**–**c’**) Staining of larval (**a**–**c**) and pupal (**a’**–**c’**) ovaries from *H^cwt^*, *H^WA^* and *H^NN^* homozygous females as indicated. Primordial germ cells were labelled with Vasa antibodies (magenta, arrows), and cell structures with Hts antibodies (green), directed against an Adducin-like protein associated with the plasma membrane cytoskeleton. Size standard, 100 µm. (**d**–**f’**) Ovaries at 3 days (**d**–**f**) versus 6 days (**d’**–**f’**) age from adult females of the indicated genotype, stained with DAPI to visualize nuclei. Arrowheads point to rudimentary ovaries in the mutants. Size standard, 200 µm. (**g**–**i**) DAPI-stained ovariole of the indicated genotype. Arrow points to stalk cells connecting the ovarioles. Size standard, 100 µm. (**j**–**l**) Numerical analysis of ovaries from homozygous females of the indicated genotype. (**j**) Counts of ovarioles (n, 25 females), (**k**) of follicles per ovarioles (n, shown above), (**l**) number of stalk cells separating follicles in respective ovarioles (n, shown above). Box blot limits indicate the 25th and 75th percentiles, whiskers show standard deviation, center line shows the median and center dot the average; outliers are indicated by dots. ANOVA for multiple comparisons according to Dunnett’s test relative to *H^cwt^* was applied (*** *p* ≤ 0.001).

As no offspring were observed from the *H^WA^* and *H^NN^* homozygotes, fertility was investigated more systematically. Homozygous flies were crossed to wild-type Oregon R in either orientation. Any larval offspring was considered as fertile and only complete absence as sterile. It turned out that females of either allele were fertile, although fertility was strongly impaired with very few larvae arising, whereas male sterility was complete. Inspection of female larval or pupal gonads from the *H^WA^* and *H^NN^* homozygotes, however, did not reveal conspicuous aberrations compared to the *H^cwt^* control. For example, the number of vasa-positive primordial germ cells in larval and pupal gonads, which eventually give rise to the germline stem cells in the female ovary, appeared largely normal (Figure 6a–c). In fact, the number of ovarioles in adult ovaries appeared significantly increased (Figure 6j). Oocyte maturation, however, was disturbed. The developing ovaries remained small and degenerate in aging females (Figure 6d–f’, Supplemental Figure S5). Only few oocytes developed into mature eggs, indicative of a failure in vitellogenesis (Supplemental Figure S5). Moreover, ovarioles contained significantly fewer egg chambers (Figure 6k). As expected for a loss of H activity, we noted a significant increase in the number of stalk cells that connect the egg chambers, which represents a typical Notch gain of function phenotype (Figure 6g–i,l) [69,70,71].

The male mutant gonads, however, revealed no gross developmental defects apart from a conspicuous swelling of the testis tip in the *H^WA^* mutants, suggesting an accumulation of germline cells (Supplemental Figure S6h). Overall, larval testes appeared normal with plenty of vasa-positive primordial stem cells forming that were also present in the testis of the adult males (Supplemental Figure S6a–f). The testicles contained the relevant structures, i.e., the testis, the seminal vesicle including mature sperms, accessory glands and the ejaculatory duct (Supplemental Figure S6g–i). Presumably, male sterility is primarily a consequence of defective locomotor activity, as we could not observe any courtship behaviour over a period of three hours in pairwise mating approaches [43].

### 3.5. H^WA^ Affects Lateral Inhibition during SOP Selection but Not the Asymmetric Cell Division of the SOP’s Daughters in Contrast to H^NN^

**Figure 7:**
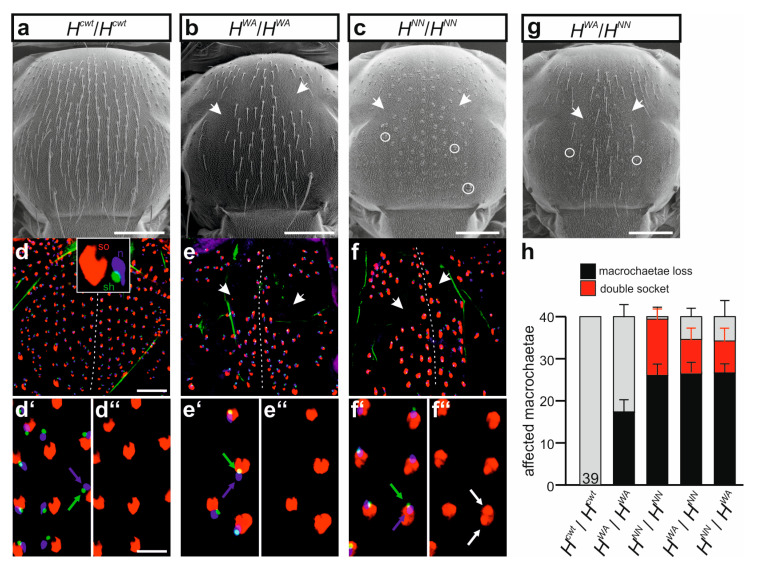
Differential activity of recessive *H* alleles with regard to bristle organ specification. (**a**–**c**) Scanning electron micrographs of homozygous females of the given genotype. Note that *H^WA^* homozygotes lack micro- and macrochaetae without a sign of shaft-to-socket transformation ((**b**), arrows point to examples), whereas the entire bristle shafts are transformed into sockets in the *H^NN^* homozygotes ((**c**), examples are encircled; baldness marked by arrows). Scale bar, 200 µm. (**d**–**f’’**) Cell type analysis of presumptive sensory organs in respective pupal thoraces. Inset in (**d**) displays cell types: socket cells (so) labelled red (anti-Su(H)), sheath cells (sh) labelled green (anti-Prospero), and neurons (n) labelled blue (anti-Elav). (**d**–**f**) Overview of the entire thorax, the presumptive midline is marked by a dotted line; areas of baldness marked by arrows (scale bar, 100 µm); (**d’**–**f’’**) respective enlargements (scale bar, 25 µm). Every sensory organ in the control (**d’**,**d’’**) as well as in *H^WA^* (**e’**,**e’’**) displays one cell type each. In contrast, sensory organs in *H^NN^* mutants frequently develop two socket cells (**f’**, white arrow in **f’’** marks one example). (**g**) Scanning electron micrograph of a heterozygous *H^WA^*/*H^NN^* female. Note the strongly reduced number of bristle organs and the mixture of sensory organs with normal appearance and double socket phenotype; examples are highlighted by circles and arrows. (**h**) Macrochaetae phenotypes determined in homo- and heterozygous adult females as indicated, compared to control (normal bristles, grey). In the heterozygotes, the allele derived from the female parent is named first. Number of total females analyzed is indicated in each column, SD is indicated.

Unexpectedly, the two alleles *H^WA^* and *H^NN^* were completely different with regard to cell fate specification during the mechano-sensory organ development. *H^WA^* homozygotes displayed losses of the entire bristle organs due to a failure of the lateral inhibition process, but no cell fate changes were observed (Figure 7a,b). In contrast, a near-complete transformation of all the outer shaft into socket cells was seen in the bristle organs of the homozygous *H^NN^* allele (Figure 7a,c). Interestingly, a further decrease in H activity, obtained by a combination with the stronger *H^LLAA^* allele, allowed the development of normal bristle organs, i.e., bristle shafts and sockets, not only in the *H^WA^*/*H^LLAA^* but also in the *H^NN^*/*H^LLAA^* trans-heterozygotes (Supplemental Figure S7a–c). The most extreme condition we could analyse was in trans over the null allele *H^attP^*. As observed earlier with other H* alleles, both *H^WA^*/*H^attP^* and *H^NN^*/*H^attP^* trans-heterozygotes developed to pharate adults that were nearly completely bald [30,33,39,54]. However, in contrast to *H^NN^*/*H^attP^* where all remaining organs displayed double sockets, *H^WA^*/*H^attP^* pharate adults still developed some apparently intact bristles (Supplemental Figure S7d–f).

Subcutaneous analyses of the developing bristle organs in pupae showed the presence of the inner cell types, i.e., the precursors of neuron and sheath cell, as well as the outer socket cell [12,39]. We noted that all prospective bristle organs contained the expected inner neuron and sheath cells in the developing pupae of the control as well as in the homozygous mutant *H^WA^* or *H^NN^* pupae (Figure 7d–f’’). In other words, cell fate transformations were restricted to the outer cell types: all prospective shaft cells were transformed to socket cells in pupal nota of the *H^NN^* homozygotes (Figure 7f–f’’), whereas no such transformation was seen in either the *H^cwt^* control or the *H^WA^* mutant (Figure 7d–e’’). The inter-allelic combination of *H^WA^* and *H^NN^* displayed a mixed phenotype, with both bristle loss and cell fate transformation, independent of the orientation of the cross (Figure 7g,h).

**Figure 8:**
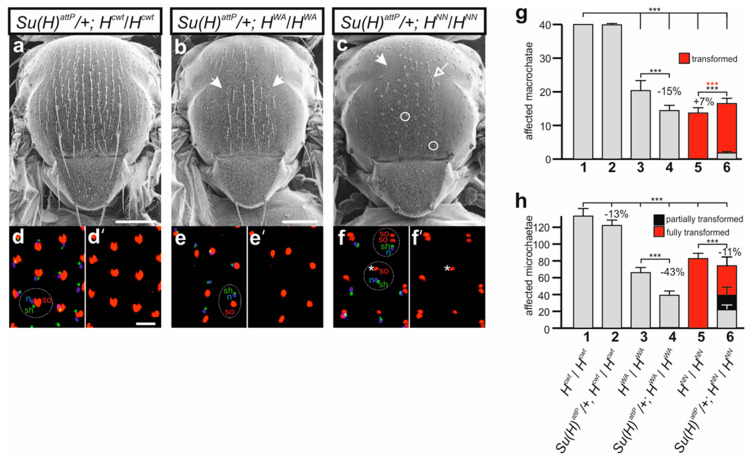
Influence of the *Su(H)* mutation on bristle phenotypes in *H^WA^* and *H^NN^* homozygotes. (**a**–**c**) Representative scanning electron micrographs of female flies of the given genotype. (**b**,**c**) Note the enhanced loss of microchaetae in the mutants (arrows point to examples). (**c**) In combination with *H^NN^* homozygotes, *Su(H)^attP^*/+ allows development of apparently normal bristle organs (open arrow points to an example) despite a profound shaft-to-socket transformation (examples are encircled). Scale bar, 200 µm. (**d**–**f’**) Cell type analysis of prospective sensory organs in respective pupal thoraces. Socket cell precursors labelled red (anti-Su(H)), sheath cell precursors (sh) labelled green (anti-Prospero), and presumptive neurons (n) labelled blue (anti-Elav). Examples of one organ each are encircled and labelled (**d**–**f**). Control *H^cwt^* (**d**,**d’**) as well as *H^WA^* (**e**,**e’**) display one cell type each, whereas many, but not all (asterisk), sensory organs in *H^NN^* mutants develop two socket cells (example encircled with dashed line). Enlargements are shown, scale bar, 25 µm. (**g**) Quantification of affected macrochaetae in the homozygous *H^cwt^*, *H^WA^* and *H^NN^* females as well as in the combinations with *Su(H)^attP^*/+. Note shaft-to-socket transformations only in *H^NN^* combinations. Genotypes 1–6 are listed in (**h**). (**h**) Microchaetae phenotypes determined in the given mutant combinations 1–6 as indicated (n = 10 females). Microchaetae located on the scutum in the area between the four dorsocentral bristles were counted and classified as normal (grey), fully (red) or partially (black) transformed (see Figure S8). (**g**,**h**) ANOVA for multiple comparisons according to Dunnett’s approach relative to *H^cwt^* and the respective homozygous *H* allele was applied for the statistics (*** *p* ≤ 0.001).

We next addressed the question of whether the bristle phenotypes of *H^WA^* and *H^NN^* could be ameliorated by a reduction of the *Su(H)* gene dose as described earlier [37,72,73,74,75]. In fact, mutations in the *Su(H)* gene were originally identified by their ability to suppress the bristle loss of *H* mutants [72,73]. In accordance with earlier reports, a suppression of the bristle loss was observed in the doubly heterozygous combination of *Su(H)^attP^*/+ with *H^attP^*/+ as well as with *H^FA^*/+ and *H^LLAA^*/+, affecting both macro- and microchaetae (Supplemental Figure S8) [36,72,73,74,75]. Most strikingly, the loss of one *Su(H)* gene copy completely abolished the typical shaft-to-socket transformations (Supplemental Figure S8) [36,74]. The nearly wild-typical phenotypes of *H^WA^*/+ and *H^NN^*/+ heterozygotes did not allow any further conclusions on the suppressive effect of *Su(H)* downregulation (Supplemental Figure S8). The picture was quite different when combining the respective homozygotes *H^WA^* and *H^NN^* mutants with the heterozygous *Su(H)^attP^*/+ allele. We noted an apparent enhancement of the bristle loss (Figure 8a–c), which was underscored by a quantification of the bristle numbers. Microchaetae numbers dropped significantly in both combinations, whereas macrochaetae were markedly lost in the *Su(H)^attP^*/+; *H^WA^* flies (Figure 8g,h). In the combination of heterozygous *Su(H)^attP^*/+ and homozygous *H^NN^*, however, we observed a reversion of the cell fate changes, i.e., formation of normal bristle shafts instead of double sockets (Figure 8c). The suppression of the transformation was also observed in the developing pupal nota, where some prospective bristle organs displayed the normal set of neuron, sheath cell and a single socket cell (Figure 8f,f’). The *Su(H)^attP^* allele had no influence on the remaining bristle organs developing in the *H^WA^* pupae, nor in the control combination (Figure 8d–e’).

## 4. Discussion

### 4.1. The Three Novel H Alleles Form an Allelic Series According to Their Residual Su(H) Binding Capability

Using genome engineering, we have generated three novel *H* alleles specifically affecting the H-Su(H) interaction to different grades. This way, we have added to the available pool of *H* alleles, many of which, however, are not well characterized in molecular detail. The major role of H is the silencing of Notch target genes. To this end, H promotes the assembly of a repressor complex by binding and connecting Su(H) with the general corepressors Groucho (Gro) and C-terminal binding protein (CtBP) [23,46,58,76]. In addition, H is important for Su(H) nuclear entry and stability [27,28,29]. Mutations in H may affect any of the mentioned aspects, (1) binding to Su(H) as outlined in this work, (2) binding to either corepressor Gro or CtBP (for example *H*^22^) [54,77], (3) nuclear translocation (for example *H^NN^*) [27], or (4) its own stability and that of Su(H) [28,29]. The consequence of any class of mutation is a reduction in H-Su(H) repressor complexes, resulting in an increase in Notch signaling activity. The resultant phenotypes are hence typical for a Notch gain of function.

Accordingly, the three new alleles show the typical *H* characteristics as expected: the mutants are recessive-larval to pupal-lethal and the heterozygous adults display dominant phenotypes, i.e., thinning and gaps in the longitudinal wing veins, and loss of micro- and macrochaetae as well as shaft-to-socket transformations in the mechano-sensory organs. The degree of these characteristic changes, however, allowed us to generate a phenotypic series with *H^FA^* being nearly a null allele, *H^LLAA^* an intermediate allele and *H^WA^* the weakest allele of the three with occasional adult flies emerging. This phenotypic series matches the residual Su(H) binding capacity as determined by isothermal titration calorimetry ITC [25]. The ITC experiments revealed a 240-fold reduction in the binding of Su(H) to the *H^FA^* mutant peptide and an around 12-fold drop in the binding to the *H^WA^* mutant peptide. The *H^LLAA^* double mutant was not assayed; however, each single LA mutation in a *H^LA^* mutant peptide affected Su(H) binding 5–10-fold; i.e., the binding capability of the double mutant is expected to be even further reduced [25].

Whereas the mutational analyses of this work allowed us to discriminate between the three novel alleles, *H^FA^*, *H^LLAA^*, *H^WA^*, our earlier experiments using overexpression analyses and cell-based reporter assays did not [32]. Overexpression during eye or wing development induced phenotypes that resembled those induced with *H^LD^*, known to completely lack Su(H) binding [24,25,28,32]. Likewise, a local induction of either of the *H* mutant variants was insufficient to prevent Notch target gene expression in vivo, in contrast to a wild-type H transgene [32]. Apparently, all three mutants were similarly strongly impaired forming repressor complexes, despite the differences measured by ITC [25]. Subtle phenotypic differences, however, reflected the ITC data. Residual protein binding, however, was uncovered in a combined overexpression of the *H* mutant variants together with Su(H). In this context, abundant repressor complexes are formed that very strongly downregulate Notch activity, causing extreme phenotypes [24,46]. In this context, any of the three mutant constructs induced phenotypes similar to a wild-type *H* construct in contrast to the binding-deficient *H^LD^* [32]. These results demonstrate the ability of the H* mutant proteins to bind to Su(H) protein if present in similarly high abundance, whereas little binding is observed when Su(H) protein levels are low. In the wild-type scenario, presumably Su(H) is already bound by the wild-type H protein which cannot be displaced by the mutant variants based on their constrained binding ability, despite being present in excess. Overall, these results uncovered the importance of the stoichiometry of H and Su(H) proteins within the cell. In the newly generated mutant H* alleles presented here, H and Su(H) expression is normal. Hence, their phenotypes directly reflect the residual Su(H) binding capacity.

### 4.2. H^WA^ and H^NN^ Homozygotes Display Multi-Morbid Phenotypes

As described earlier for the recessive *H*^22^ allele, *H^WA^* and *H^NN^* homozygotes are short-lived [33]. As the strong *H* mutant alleles die during larval and pupal stages, we assayed food finding and uptake in the larvae, which, however, appeared unchanged. Presumably, the mutants die not of starvation. However, we cannot exclude lasting problems in nourishment, because Notch activity plays important roles throughout the development of the midgut as well as in intestine homeostasis. Notably, the generation of midgut progenitors, stem cell maintenance and the formation of the absorptive enterocytes relies on Notch activity (reviewed in [78,79,80]). In addition, Notch directly regulates glycolytic genes [81]. Malnutrition might explain the sluggish appearance of the mutants, in accord with the large variety of behavioral defects apart from the described extreme wing and bristle phenotypes. The homozygotes were hesitant moving or climbing, and were completely flightless. These phenotypes, however, could also reflect defects in the locomotor system, as the development of either the musculature or the central and peripheral nervous system as well as the motor circuits relies on repeated Notch signaling activity (reviewed in [6,82,83]). Apart from its role in neurogenesis and neural stem cell determination, Notch activity is instrumental for neuronal identity, for example, neurotransmitter identity, in the larval as well as in the adult central nervous system [84,85,86,87,88,89]. In addition, Notch activity plays important post-developmental roles in the brain [90,91]. Hence, defective brain function or control of locomotion are to be expected in the homozygous *H* mutants which represent a Notch gain of function background, easily explaining the aberrant behaviors. In this respect, the lack of courtship behavior in the mutant males is not surprising, and is in accord with the complete sterility of the *H^WA^* and *H^NN^* homozygotes. Additional defects in testis or sperm development, however, cannot be excluded, as the male flies did not prove their talents. Clearly, ovaries displayed various abnormalities; notably, vitellogenesis was incomplete. The strongly reduced number of mature eggs observed in the mutant *H* females correlates well with the impeded fertility. Again, since Notch activity is required at several steps of gonadal development of both males and females, respective defects are to be expected (reviewed in [9]). To summarize, the morbidity of the *H^WA^* and *H^NN^* homozygous mutants cannot be explained by a singular cause but rather by the general increase in Notch activity, resulting in a variety of phenotypes in many different organs, reflecting the pleiotropic roles of Notch during development and cellular homeostasis.

### 4.3. The Phenotypic Differences between H^WA^ and H^NN^ Homozygotes with Regard to Cell Type Specification Point to a Different Availability of Su(H)

Both, *H^WA^* and *H^NN^* homozygotes displayed a similarly strong loss of mechano-sensory organs, with slightly less macrochaetae in the *H^NN^* homozygotes versus slightly less microchaetae in the *H^WA^* homozygotes, each compared to the other (see Figure 7h, Figure 8g,h and Figure S8c). The two alleles appeared quite similar overall, albeit *H^WA^* might retain slightly more H activity based on survival rates and wing phenotypes (Figure 5a and Figure S2). A downregulation of H activity entails elevated Notch signaling activity. Accordingly, mechano-sensory organs fail to develop because of the increased lateral inhibition affecting the sensory organ precursor cell (SOP). SOPs are selected from proneural clusters by lateral inhibition involving Notch signaling. In a wild-type situation, signals from the SOP activate Notch in the adjacent cells, which turn on the Notch target genes of *Enhancer of split complex (E(spl)-C)*, conferring epidermal fate (Figure 1a) (reviewed in: [3,4,5,23]). At the same time, the SOP must be protected from basal Notch signals. This involves the H-Su(H) repressor complex including the corepressors Gro and CtBP, as well as cis-inhibition of the Notch receptor by its ligands [59,92,93,94,95]. In fact, spurious activity of *E(spl)-C* genes within the SOP causes its extinction [94]. We noted a typical pattern of bristle loss, i.e., the central rows of microchaetae usually remained intact, whilst primarily the fifth row was lost and balding affected anterior and lateral regions (Figure 7 and Figure S7). The loss of rows roughly corresponds to their emergence during pupal stages [96]. The different sensitivity of bristle rows to loss of H activity might be explained by the self-organizing process of microchaetae arrangement, involving a dynamic balance between proneural factors and E(spl)-C proteins [97,98]. Striking differences, however, were observed regarding the cellular composition of the bristle organs.

Whereas both *H^WA^* and *H^NN^* mutants were equally impeded in protecting the SOP from basal Notch activity, they were completely different with regard to the subsequent cell type specification. *H^NN^* mutant flies displayed the characteristic double socket phenotype, whereas *H^WA^* developed normal bristles including shaft and socket cell (Figure 7b,c). The double socket phenotype of *H^NN^* resulted from a cell fate change rather than from an extra division of the SOP as uncovered by the analysis of the respective precursor cells in pupae (Figure 7f–f’’). An extra cell division of the SOP could give rise to extra external cells, and may result from a disturbance of the G2 phase arrest, which is a prerequisite for the subsequent asymmetric division [99]. We can also exclude a transformation of the inner to the outer cell sublineage as observed upon downregulation of the genes *escargot* and *scratched* [100]. In the inner cell lineage, both factors serve to maintain the inhibition of Notch target genes essential for neural precursor identity [100].

It is well established that the unequal division of the SOP gives rise to the outer (pIIa) and the inner (pIIb) cell sublineages (Figure 1a). This binary cell fate decision is under the regulation of Notch signaling activity, and manipulation of this activity induces a collapse of the asymmetry (reviewed in [101]). Briefly, both SOP daughters are fated towards outer cell fate in the case of a reduction in Notch activity, whereas they both develop into inner cell types in consequence of a gain in Notch activity. Apparently, Notch signaling is activated within the pIIa cell by signals derived from the pIIb and perhaps surrounding epidermal cells [101,102]. Directionality of Notch signaling is ensured by the inhibitory activity of Numb via endocytic sorting of Notch within the pIIb, resulting in an asymmetric availability of Notch receptor molecules at the pIIb cell surface (reviewed in [101,103]). Interestingly, specification of the SOP daughters pIIa/b is undisturbed in either of the two *H* mutants. The subsequent cell division of the pIIa into shaft and socket precursor cells is likewise asymmetric, and is mediated by differential Notch signaling activity under the regulation of Numb and Hairless (reviewed in [15,101]). Again, two equal cells arise if Notch signaling is not unidirectional, i.e., two shaft cells develop in response to a downregulation of Notch activity versus two socket cells, if Notch activity is in excess.

Indeed, shaft cell fate can be considered a default fate, as it is signal-independent [15,104]. Accordingly, the socket cell requires Notch signals to repress the shaft fate. Shaft cell fate relies on the expression of the Pax2-type transcription factor shaven (sv) [105,106]. Within the socket cell, *sv* expression is inhibited by Su(H) together with Sox-15 [104,105]. The subsequent induction of an autoregulatory feedback loop results in a strong increase in Su(H) expression within the socket cell, which is necessary for socket cell differentiation and function [104,107]. Along the lines of the lateral inhibition mechanism, it has been proposed that the shaft cell crucially depends on the basal inhibitory activity of the H-Su(H) repressor complex. The H-Su(H) repressor complex is required for preventing *sv* repression by spurious or basal Notch signals that otherwise would start the Su(H) autoactivation program resulting in a shaft-to-socket fate change [58,94,104]. Accordingly, genetic combinations of *H* and *sv* mutants strongly enhance the formation of double sockets at the expense of bristle shafts [53]. In the absence of either repressor component, a transformation of shaft into socket cell fate is very likely by inappropriately activating Notch pathway target genes. This is apparent in a background of reduced H activity like in the *H*/+ heterozygous condition, where double sockets are formed (see e.g., Figure 3a). The shaft-to-socket transformation, however, requires Notch signaling activity mediated by Su(H). Accordingly, a reduction in *Su(H)* gene copies can suppress the *H*/+ phenotype as observed in the double heterozygotes (see Supplemental Figure S8) [36,72,73,75]. Apparently, *H*/+ represents a sensitized background regarding socket fate, which is enhanced by reducing repressor capacity; however, it is alleviated by weaker Notch signaling activity. For example, the double socket phenotype in *H*/+ is rescued by a downregulation of Notch activity induced by Numb overexpression within the pIIa cell lineage [39].

**Figure 9:**
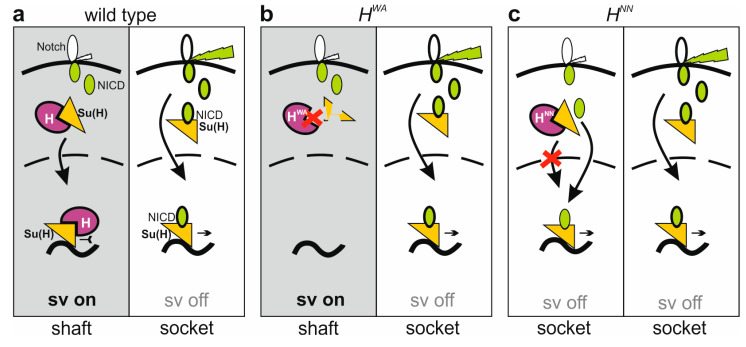
Highly simplified model of shaft fate specification. For simplicity, only relevant players in single copy are shown. (**a**) Left: By default, cells are fated towards shaft due to the activity of shaven (sv). Spurious Notch signals are inhibited by the H-Su(H) repressor complex. Right: upon activation (green bolt) of the Notch receptor, NICD is released to form the NICD-Su(H) activation complex, igniting Su(H) autoactivation and repression of *sv*, eventually driving socket cell differentiation. (**b**) Left: Due to impaired binding, H^WA^-Su(H) complexes are instable, as is Su(H). Limited availability of Su(H) restrains activator complex formation despite spurious Notch activation. As *sv* expression is not inhibited, shaft fate remains by default. Right: socket fate is unaffected in the *H^WA^* mutant. (**c**) Left: *H^NN^*-Su(H) complexes are impaired in nuclear entry, and spurious Notch activity is not repressed. Instead, NICD-Su(H) activator complexes are formed and start a repression cascade of *sv*, resulting in a shaft-to-socket fate transformation. Right: socket fate is unaffected in the *H^NN^* mutant.

H activity is even further reduced in *H* homozygous mutants, reflected by the near complete transformation of shafts into sockets in *H^NN^* or *H*^22^ alleles (Figure 7) [30,33]. Why do *H^WA^* homozygotes then develop bristle shafts? One might speculate that *H^WA^* retains sufficient H activity for shaft formation. However, bristle shaft development does not simply correlate with the level of H activity. For example, we observed normal bristle shafts in both the *H^WA^*/*H^LLAA^* and the *H^NN^*/*H^LLAA^* combinations, despite the fact that the *H^LLAA^* allele affects H activity even more strongly (Supplemental Figure S7b,c). In all respects, *H^LLAA^* is a rather strong *H* allele retaining little H activity, and is clearly stronger than *H^NN^* or *H^WA^* (Figure 3 and Figure 4). Accordingly, bristle organ loss increased overall in the *H^WA^*/*H^LLAA^* or *H^NN^*/*H^LLAA^* heteroallelic combinations, yet none displayed more double sockets (Supplemental Figure S7b,c). Apparently, in *H^WA^* homozygotes presumptive shaft cells do not receive the transformant Notch input, despite a strong gain in Notch activity. We can compare the three alleles, *H^WA^*, *H^NN^* and *H*^22^ at the molecular level: while *H^WA^* affects the binding to Su(H), *H^NN^* impairs nuclear H translocation and *H*^22^ impairs corepressor recruitment. Both, *H^NN^* and *H*^22^ bind normally to Su(H) (Figure 2a) [47], i.e., are able to form H-Su(H) protein complexes in the cytoplasm. We have shown earlier that the Su(H) protein is present at very low levels within the cytoplasm; however, it is stabilized by complexing with either H or NICD [28,29]. Hence, in the *H^NN^* and *H*^22^ homozygous background, Su(H) protein levels would be similar to the wild-type background, whereas in *H^WA^* homozygous cells, the total level of Su(H) protein might be reduced, as fewer H-Su(H) complexes are formed due to the impeded H^WA^-Su(H) binding. In the *H^WA^*/*H^LLAA^* or *H^NN^*/*H^LLAA^* heteroallelic combinations, even fewer H^LLAA^-Su(H) complexes are expected, lowering Su(H) availability even further. As NICD is able to outcompete H from H-Su(H) complexes [24], lowered levels of H-Su(H) complexes would translate into a lowered availability of Su(H) for the formation of activator complexes as a consequence (Figure 9). This model could explain why spurious or basal Notch signals are insufficient to initiate a shaft-to-socket transformation.

Apparently, shaft and socket cell fates rely on a delicate balance between H-Su(H) repressor and NICD-Su(H) activator complexes, whereby their formation depends on the respective affinities, influencing the availability of Su(H) protein within the cell.

## 5. Conclusions

Genome engineering allowed us to establish three novel *H* alleles with mutations predicted to specifically affect the interface of the H-Su(H) protein complex. Resultant developmental defects were in accord with the expectations, reflecting the residual in vitro binding of respective short H peptides to Su(H), thereby confirming the proposed structure of the H-Su(H) protein complex. Apparently, the H-Su(H) interface involves several amino acid contacts in combination, none of which alone being sufficient for the binding. This notion was known for the Su(H) side, but also holds true for the H side as demonstrated in this work. Previous H data comprised only a disruptive mutation (*H^LD^*), disallowing any further conclusions on the H contact sites within the repressor complex [24,30]. Notably, our previous overexpression analyses did not permit discriminating between the three alleles: in the one context, they all appeared non-functional, while in the other they all matched the wild type [32]. Hence, the current work adds to our understanding of the basis for the H-Su(H) interaction. Moreover, the viability of the homozygous *H^WA^* and *H^NN^* alleles enabled us to provide a first in-depth analysis of the pleiotropic phenotypes caused by a strong *H* loss of function in adult flies. In addition to the well-described defects in wing venation and mechano-sensory organ formation, *H* homozygotes were short-lived and displayed several behavioral defects and sterility. The sluggishness of the adults may result from malnutrition, for example, due to intestinal defects, and/or from a failure of specific brain functions or the control of locomotion. Female and male sterility may result from aberrant ovary and testicular development, apart from the behavioral defects. These multi-morbid phenotypes certainly warrant a more comprehensive investigation in the future.

Finally, the differential activity of the two alleles *H^WA^* and *H^NN^* regarding bristle organ development uncovered an exquisite sensitivity of shaft cell fate specification for the H-Su(H) protein affinities. Earlier models highlighted the stoichiometry of H and Su(H) proteins as a fundamental parameter for binary cell fate decisions [15]. Our work now provides evidence that, rather, the respective protein affinities are important as they affect the availability of Su(H) for NICD binding. NICD-Su(H) activator complexes inhibit default shaft cell fate specification. As the binding to H affects Su(H) stability, the lower affinity of the *H^WA^* allele to Su(H) may reduce the availability of Su(H), and hence the formation of NICD-Su(H) activator complexes, thereby allowing shaft cells to form. Whether binary cell fate decisions in mammals are similarly regulated by the availability of the CSL homologues is less clear. The regulation of the stability of mammalian CSL homologues, however, has been shown to influence normal as well as tumour development in several instances [108,109,110]. In conclusion, our work provides important further insight into the regulation of the Notch signaling pathway. As the fine-tuning of Notch activity is also critical to both development and disease in mammals, we hope that our work opens the avenue for further investigations with medical implications in the future.

## Data Availability

The original contributions presented in the study are included in the article and Supplementary Material; further inquiries can be directed to the corresponding author.

## Supplementary Materials

The following supporting information can be downloaded at: https://www.mdpi.com/article/10.3390/genes15050552/s1, Figure S1: Uncropped Western blot; Figure S2: Wing phenotypes of homozygous *H* mutants; Figure S3: *H^WA^* and *H^NN^* homozygotes are flightless; Figure S4: qRT-PCR report; Figure S5: Retardation of egg maturation; Figure S6: Normal testicles despite full sterility; Figure S7: Bristle defects in interallelic combinations with *H^LLAA^* and *H^attP^*; Figure S8: Influence of loss of Su(H) on the bristle phenotypes of *H* heterozygotes.
