# Supplementary material for: Novel Genome-Engineered H Alleles Differentially Affect Lateral Inhibition and Cell Dichotomy Processes during Bristle Organ Development
Source: Genes (Basel). 2024 Apr 26;15(5):552. doi: 10.3390/genes15050552 (PMC11121709; doi:10.3390/genes15050552)
Supplement: Supplementary file 1 [file genes-15-00552-s001.zip › genes-2959186-supplementary.pdf]

Supplemental Material

Tanja C. Mönch, Thomas K. Smylla, Franziska Brändle, Anette Preiss, Anja C. Nagel,

Novel genome engineered *H* alleles differentially affect lateral inhibition and cell dichotomy processes during bristle organ development

Figure S1 Uncropped Western blot

Figure S2 Wing phenotypes of homozygous *H* mutants

Figure S3 H^{WA} and H^{NN} homozygotes are flightless

Figure S4 qRT-PCR report

Figure S5 Retardation of egg maturation

Figure S6 Normal testicles despite full sterility

Figure S7 Bristle defects in interallelic combinations with H^{LLAA} and H^{attP}

Figure S8 Influence of loss of Su(H) on the bristle phenotypes of *H* heterozygotes

Figure S1 Uncropped Western blot

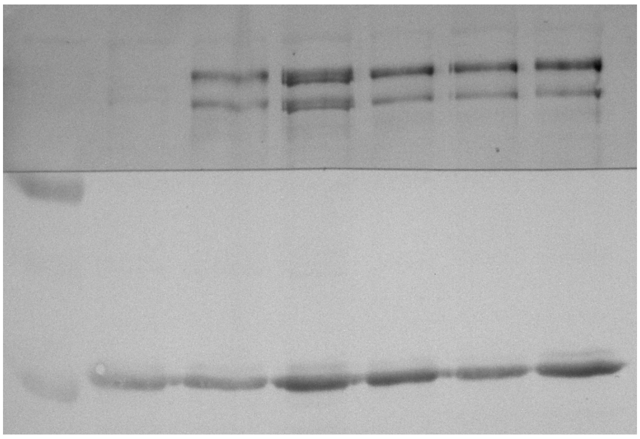

Uncropped Western blot, shown in Figure 2c. The blot was sliced after transfer and developed with antiserum against H (upper half) and beta-tubulin (lower half), respectively.

Figure S2 Wing phenotypes of homozygous *H* mutants

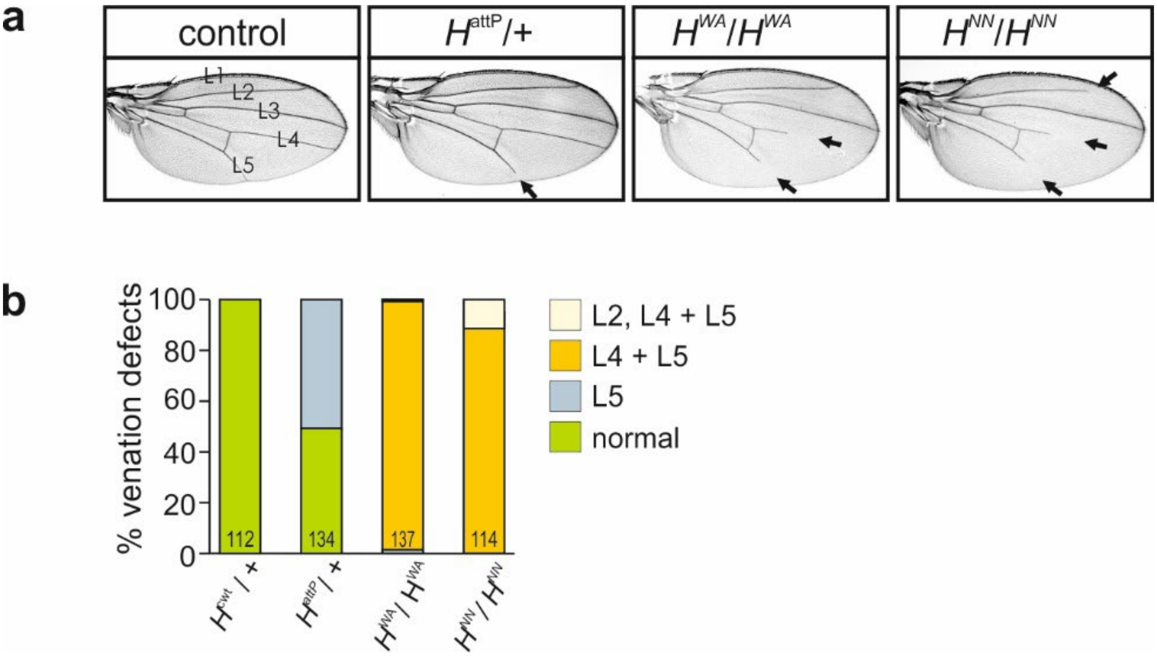

(a) Representative examples of wings from female flies of the given genotype. Longitudinal veins are labelled in the control. Note that in the mutants, longitudinal vein L5, and/or L4 and L2 do not reach the wing margin (marked by arrows). (b) Quantification of wings with indicated vein defects, given as the fraction of the total (n, indicated in each column).

Figure S3 H^{WA} and H^{NN} homozygotes are flightless

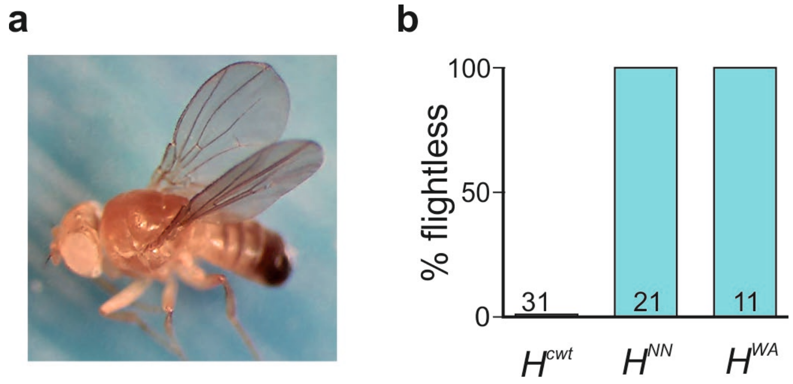

(a) Erect wing posture frequently observed in H^{WA} and H^{NN} adults: Normally, *Drosophila* flies hold their wings parallel to the ground, whereas wings are turned up in the mutant. H^{NN} male is shown. (b) In contrast to the H^{cwt} control, neither H^{WA} nor H^{NN} homozygous adults can fly in a flight assay. Number of tested adults is given in each column.

Figure S4 qRT-PCR report

Generated by micPCR version 2.12.2

Relative Quantification: HcWT_HW258A_HNLS3NES

**Method**	REST
**Efficiency**	CyclingAnalysisCalculated

**Gene of Interest**	**Efficiency**
GstD1_1	0,89
cnc_1	0,92
ewg_3	0,94
mTFB2_3	0,93

**Reference Gene**	**Efficiency**
cyp33	0,91
Tbp	0,90

**Control Group**	H_cWT
**Treatment Groups**	H_W258A; H_NLS3NES

**Gene**	**Treatment**	**Expr. Ratio**	**Std. Error**	**95% Confidence**	**P Value**	**Result**
GstD1_1	H_W258A	1,116	0,886 - 1,443	0,752 - 1,663	0,4920	No difference
cnc_1	H_W258A	0,758	0,675 - 0,850	0,640 - 0,896	0,0195	Down
ewg_3	H_W258A	0,966	0,840 - 1,179	0,711 - 1,241	0,7030	No difference
mTFB2_3	H_W258A	0,965	0,851 - 1,092	0,781 - 1,168	0,6060	No difference
GstD1_1	H_NLS3NES	1,124	0,990 - 1,274	0,855 - 1,471	0,2320	No difference
cnc_1	H_NLS3NES	0,789	0,703 - 0,908	0,656 - 0,974	0,0130	Down
ewg_3	H_NLS3NES	1,019	0,826 - 1,165	0,788 - 1,443	0,8790	No difference
mTFB2_3	H_NLS3NES	0,969	0,888 - 1,055	0,834 - 1,115	0,5665	No difference

Figure S5 Retardation of egg maturation

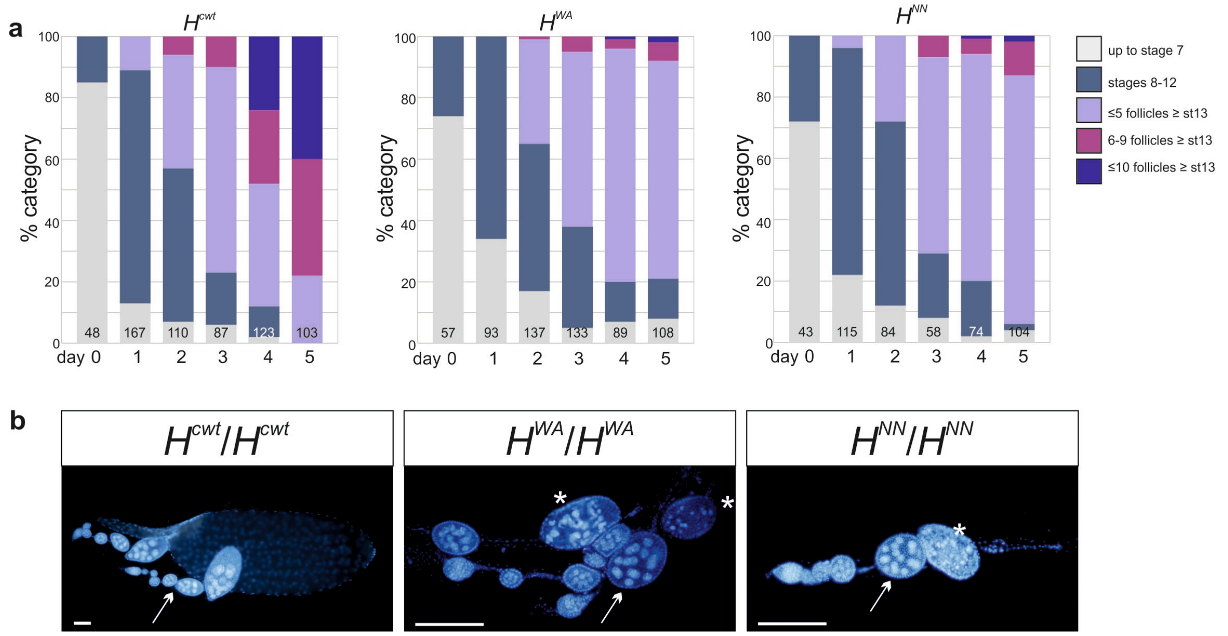

(a) Follicle development evaluated in ovaries of respective homozygous females at day 0 to 5 post eclosion as indicated (at 25°C). Five categories were chosen and presented as percentage of the total: (1) follicles developed up to stage 7, i.e. before vitellogenesis, (2) follicles reached stages 8-12, (3) up to five mature eggs per ovary, (4) 6-9 mature eggs per ovary, (5) 10 or more mature eggs (≥ stage 13). The number of ovaries analyzed is indicated in the column. (b) Ovaries stained with DAPI to label nuclei. Asterisks mark examples of deteriorating follicles as observed at times in the mutant females, but rarely in the control. Arrows point to follicles at stage 8-9. Size standard, 25 µm.

Figure S6 Superficially normal testicles despite full sterility

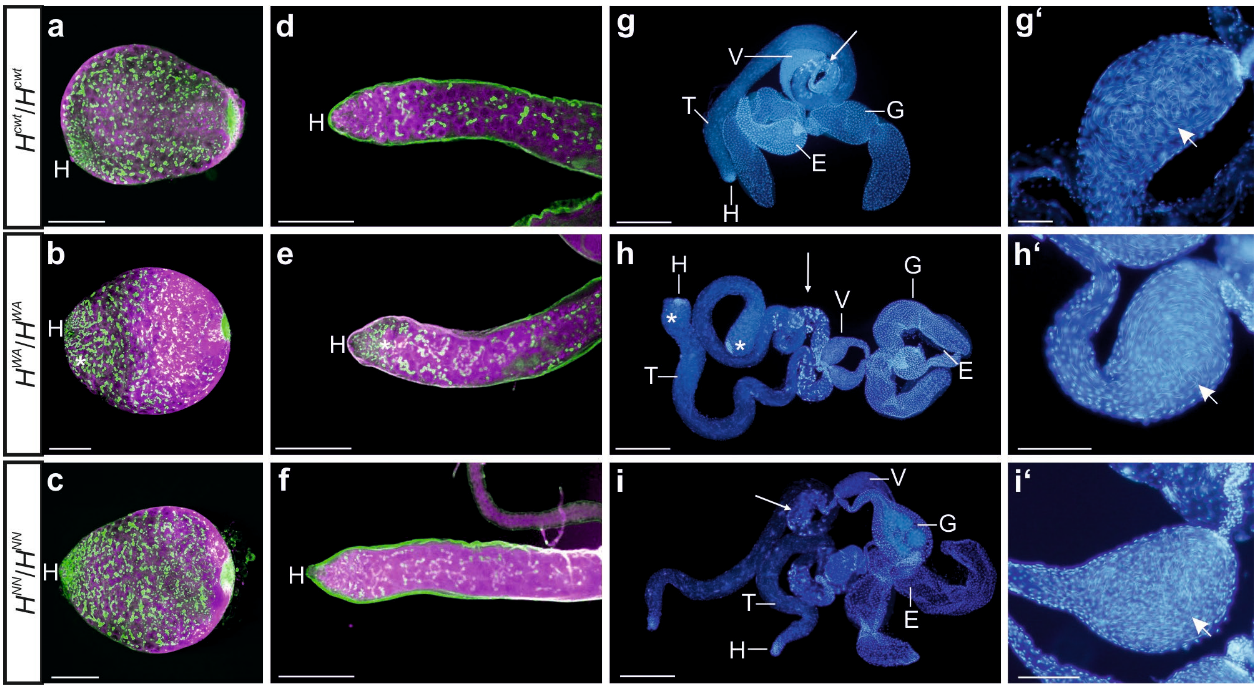

Legend to Figure S6
(a-c) Male gonads from early pupae (about 24h APF) of the given genotype stained for the primordial germ cells (magenta, anti-Vasa). Cells are outlined by detecting the Adducin-like protein Hts (Hu-li tai shao), associated with the plasma membrane cytoskeleton (green, anti-1B1). The hub (H) harbouring the stem cell niche is marked. (d-f) Apical tip of testes from adult males of the indicated genotype, stained for vasa (magenta) and 1B1 (green). H, hub. Conspicuous enrichment of Vasa-positive germline cells in H^{WA} (e, asterisk). (a-f), Size standard 100µm. (g-i) DAPI stained testicles from control and mutant males as indicated. They all contain the normal complement of testis (T), with hub (H, stem cell niche), seminal vesicle (V), accessory gland (G) and ejaculatory duct (E). Note individualizing spermatids (arrow). Conspicuous swelling of testis tips in H^{WA} (h, asterisks). Size standard, 250 µm.
(g'-i') Enlargement of seminal vesicles; mature sperms are present (arrow). Size standard, 25 µm.

Figure S7 Bristle defects in interallelic combinations with H^{LLAA} and H^{attP}

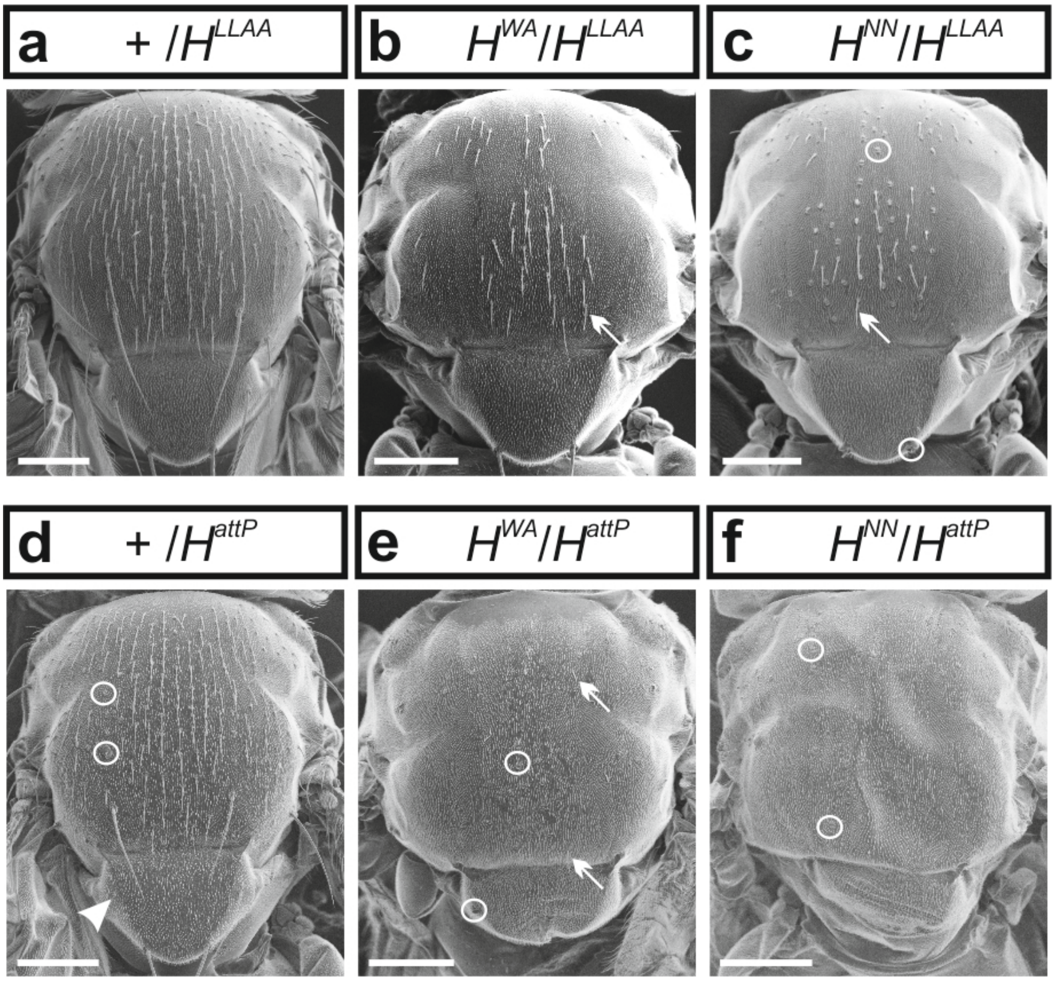

(a-f) Scanning electron micrographs of animals with the indicated genotype. (c) H^{NN}/H^{LLAA} reared at 18°C. Circles exemplify shaft to socket transformations; arrows point to examples of remaining microchaetae. Note that pharate adults are displayed except for the controls in (a,d). Occasionally, notal collapse can occur in the vacuum during scanning electron microscopy as in (f). Size bar, 200 μm.

Figure S8 Influence of loss of Su(H) on the bristle phenotypes of *H* heterozygotes

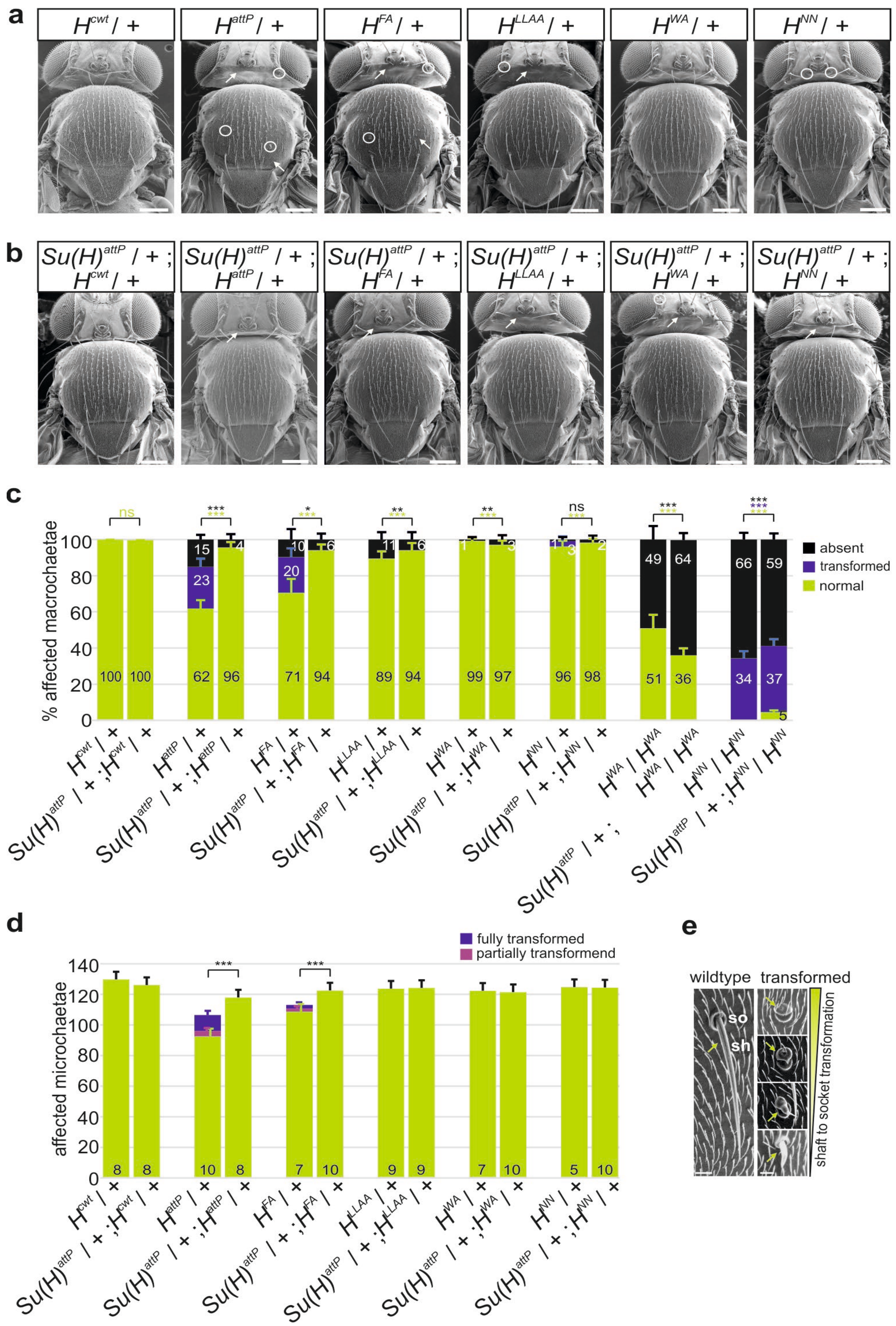

Legend to Figure S8

(a) Representative scanning electron micrographs of heterozygous *H**/+ females as indicated; arrows exemplify the loss of micro- and macrochaetae and circles the shaft to socket transformations. (b) Representative scanning electron micrographs of doubly heterozygous *Su(H)*^{attP}/+; *H**/+ female flies; the genotype is given above. Arrows exemplify the loss of macrochaetae, and the circle shows a rare partial shaft to socket transformation. Size bar, 200 µm in (a,b).

(c) Macrochaetae were classified as either normal, absent or transformed (shaft to socket transformation). The fraction of respectively affected macrochaete is indicated; columns show the average, whiskers the standard deviation (insets give respective percentage). Twenty females of each genotype were analysed. For statistical analysis, Student's T-test was applied, and is color-coded for changes of normal bristles and of bristle loss (*** $p<0.001$, ** $p<0.01$, * $p<0.05$, ns, $p\geq0.05$).

(d) Microchaetae numbers scored in the field confined by the four dorso-central bristles; columns show the average, whiskers the standard deviation. Full or partial shaft-to-socket transformation was scored as indicated. Number of analyzed females is shown in each column. For statistical analysis, Student's T-test was applied for changes in bristles numbers; only significant changes are indicated (*** $p<0.001$).

(e) Examples of the range of incomplete shaft to socket transformation. Green arrows point to (transforming) shaft. Left, normal microchaetae with socket (so) and shaft (sh). Size bar, 10µm.
